# Emergency Contraceptives Use Among Female Commercial Sex Workers in Tanzania: A Cross Sectional Study

**DOI:** 10.24248/eahrj.v9i1.830

**Published:** 2025-09-30

**Authors:** Elihuruma Eliufoo Stephano, Yohana Swebe Masubho, Yusheng Tian, Fabiola Moshi, Stephen Kibusi, Yamin Li

**Affiliations:** a Clinical Nursing Teaching and Research Section, The Second Xiangya Hospital, Central South University, Changsha, Hunan 410011, China; b Department of Clinical Nursing, School of Nursing and Public Health, The University of Dodoma, Dodoma, Tanzania; c Medical Department, Doctors Without Borders (Medensis Sans Fronteres), Dar es Salaam, Tanzania; d Department of Public Health and Community Nursing, School of Nursing and Public Health, The University of Dodoma, Dodoma, Tanzania.

## Abstract

**Background:**

Unintended pregnancies pose a significant health risk for Female Commercial Sex Workers (FCSW), a highly vulnerable population. Despite the availability of emergency contraceptives (EC) to prevent such outcomes, concerns still exist regarding potential misuse as a regular contraceptive method, and the specific factors influencing EC use among FCSWs remain underexplored. This study aimed to evaluate the prevalence and determinants of EC use among FCSWs to address this critical knowledge gap.

**Methods:**

This analytical cross-sectional study involved 326 randomly selected female commercial sex workers in Tanzania. Data were collected using a structured questionnaire created from previous literature and analyzed using SPSS version 29. Given the sensitive nature of this population, detailed ethical procedures were followed to ensure voluntary participation and confidentiality. Our sampling approach utilized community-based outreach rather than a formalized registry.

**Results:**

The study found that a significant majority of the respondents (64.4%) had never used EC. Multivariate logistic regression analysis identified several key factors independently associated with EC use. Education level showed an inverse relationship, with those having ordinary secondary education (AOR=0.37, 95% CI=0.16–0.88, *P*=.024) and college-level education (AOR=0.13, 95% CI=0.04–0.47, *P*=.002) being significantly less likely to use EC compared to those with no formal education. Furthermore, a history of drug use was strongly associated with a reduced likelihood of EC use (AOR=0.35, 95% CI=0.19–0.61, *P*<.001). Conversely, prior awareness of emergency contraceptives emerged as a powerful predictor of use, with those who had ever heard about EC being substantially more likely to use them (AOR=6.20, 95% CI=3.44–11.17, *P*<.001).

**Conclusion:**

The use of emergency contraceptives among FCSW is still low for various reasons including educational attainment, and drug use. While awareness is a strong facilitator of EC use, higher education levels surprisingly showed an inverse relationship. This suggests that interventions are needed to address the unique barriers faced by this vulnerable group, including misconceptions among the educated and the compounding challenges posed by drug use. Several approach combining comprehensive education, integrated health services, and sustained awareness campaigns is essential to improve EC access and utilization for FCSW.

## BACKGROUND

Female Commercial Sex Workers (FCSW) face a constant risk of abuse from clients and the community, contributing to a high rate of unintended pregnancies globally.^1^ Despite advancements in contraceptive efficacy, sex workers experience a disproportionate number of unintended pregnancies (UIPs) compared to women who do not sell sex.^2^ Between 50% and 65% of sex workers undergo at least one termination of an unintended pregnancy, which indicates significant unmet reproductive health needs.^3^

Unintended pregnancy and HIV infection are significant occupational hazards for female sex workers.^1^ Globally, 74 million women in low and middle-income countries (LMICs) experience unintended pregnancies annually, leading to 25 million unsafe abortions and 47,000 maternal deaths each year.^4^ In Sub-Saharan Africa (SSA), the prevalence of unwanted pregnancies and abortions continues to rise.^4,5^ The region accounts for over 14 million unplanned pregnancies annually, with an overall prevalence rate of 29%.^4^ Among female sex workers in SSA, rates of non-barrier contraceptive use vary from 15% to 76%, unintended pregnancy from 24% to 91%, and abortion from 11% to 48%. Factors such as alcohol use, violence, issues with health systems, and socioeconomic problems are common across these regions.^6^ Emergency contraception (EC) can reduce the risk of unintended pregnancy by over 95% if taken within 72 hours of unprotected sexual intercourse.^4^ However, awareness and use of EC among women of reproductive age in SSA remain limited despite its availability, safety, and efficacy.^4,6^

The lowest reported prevalence of current EC use among FCSW was 3.1% in Uganda, while the highest was 21% in China.^7^ Lifetime EC use among FCSW has ranged from 2.4% in India to 50.7% in Nigeria, with a median of 27.5% reported in Swaziland.^7,8^ Low contraceptive use and a high burden of unintended pregnancy led to poor reproductive outcomes and avoidable mother-to-child HIV transmission risks among FCSW.^9^

In Tanzania, there is an alarming surge in the misuse of ECs among reproductive-aged females, particularly younger girls and women.^10^ This misuse includes using ECs as regular contraceptives, which can increase the risk of contraceptive failure and adverse health events.^11^ Despite the significant increase in EC consumption, a study found that majority of Tanzanian FCSW lack basic knowledge on the proper use of ECs.^12^ Studies in Tanzania reveal that FCSW in Dar es Salaam have a high HIV prevalence of 15%, which is 2.5 times the national average.^13^ Another survey in one region of Tanzania shows an even higher HIV prevalence of 40.9% among FCSW, 8.1 times higher than the general population.^14^ Unmet contraceptive needs among FCSW are common and mostly due to legal implication and nature of the work, leading to high rates of unintended pregnancies and terminations.^15^

Sex work is criminalized under Tanzanian law. Loitering for the purpose of prostitution on the mainland carries a three-month prison penalty. The Penal Code of Tanzania and the Penal Decree Act of Zanzibar also criminalize “carnal knowledge against the order of nature.”^16^ These laws deny sex workers the right to bodily autonomy and the choice to make decisions about their lives, profession, and bodies.^17^ Sex workers in Tanzania may also be arrested for not conforming to gender norms.^18^ The criminalization of sex work leads to discrimination and social isolation, making FCSW hesitant to disclose their occupation to healthcare providers due to fear of stigma and persecution.^3^ This legal framework impacts their ability to access essential health services.^19^

Female commercial sex workers in Tanzania face a high burden of unintended pregnancies and poor reproductive outcomes due to low contraceptive use.^15^ Despite reported awareness of emergency contraceptives, a significant portion of FSWs do not use them, indicating a critical gap between knowledge and practice.^7^ The criminalization of sex work in Tanzania further exacerbates these issues by creating barriers to healthcare access and limiting reproductive agency.^20^ This study aims to assess the use of emergency contraceptives among female commercial sex workers in Dodoma city, Tanzania, to understand the problem in preventive measures and emergency contraception. The rationale for this study is to generate more specific information that can guide the development of effective interventions, contraceptive counseling guidelines, and theories tailored for high-risk populations like sex workers, ultimately improving reproductive health outcomes, preventing unintended pregnancies, and understanding the specific determinants of emergency contraceptive use.

### Theoretical Framework

Connell's theory of gender and power laid the foundation for our investigation of how sex workers use contraception. We paid particular attention to factors impacting contraceptive usage and contraceptive decision-making in FCSW.^21^ Connell recognized three components in the theory of gender and power: cathexis, the sexual division of labour (economic exposures and socioeconomic risk factors), and physical and/or sexual violence and substance misuse. These concepts are crucial for comprehending how men and women relate to one another regarding social standards and emotional ties. A power or agency imbalance favouring the male spouse may negatively affect the female partner's reproductive health in any of these three categories.^21^

## METHODS

### Study Design and Setting

This analytical cross-sectional study was conducted between March and September 2019 in Dodoma City, Tanzania. The city, which is the capital of Tanzania and one of its fastest-growing urban centers, was strategically chosen for this research due to its large and rapidly expanding young population. According to the most recent census, Dodoma City has a population of 3,085,625 residents.^22^

### Study Participants

The study population comprised Female Commercial Sex Workers (FCSW) in Dodoma City who voluntarily agreed to participate. Eligibility for participation required respondents to be female, at least 18 years old, and to have engaged in sex work (receiving money or gifts in exchange for sex) at least three times within the preceding six months. Individuals who were seriously ill or had severe mental health problems were excluded from the study. Following informed consent, eligible participants completed an interviewer-administered, standardized questionnaire that collected data relevant to the study's objectives.

### Sample Size

A single population proportion formula was employed to calculate the sample size, assuming a 50% prevalence of emergency contraception use among FCSW, a 95% confidence level, and a 5% margin of error. The prevalence of 50% was assumed because there was no previous study established the prevalence of EC use among FCSW. This calculation yielded a required sample size of 384 participants. However, after verifying eligibility and ensuring full participation, 326 individuals were ultimately enrolled in the study.

### Sampling Procedure

For the sampling procedure, this study implemented a multi-stage approach to minimize selection bias and enhance representativeness. From a comprehensive list of Dodoma City's 41 wards, 22 wards known to contain commercial sex working locations (e.g., pubs, bars, nightclubs, liquor stores, guesthouses, and other public accommodations) were randomly selected. Subsequently, ward leaders in these chosen wards provided a comprehensive list of FCSWs, including their contact details, which served as our sampling frame. The total number of study participants was then allocated to each selected ward proportionally to its estimated FCSW population size. Within each ward, individual participants for interviews were chosen using a simple random selection process facilitated by Microsoft Excel. Once potential participants were identified, trained research assistants approached them discreetly and privately, explaining the study's purpose, procedures, and the voluntary nature of participation. Emphasis was placed on ensuring confidentiality and anonymity throughout the entire process. If an FCSW expressed interest, a detailed informed consent process was conducted, outlining their rights, the risks and benefits of participation, and their ability to withdraw at any time without penalty. Only those who provided clear, informed consent were then included in the study for subsequent interviews.

**FIGURE 1:**
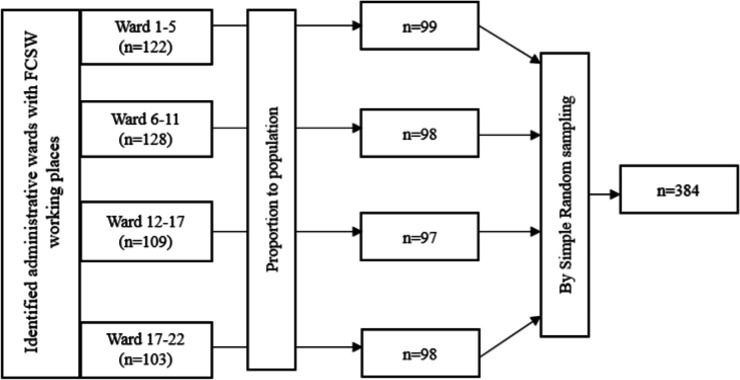
Details for FCSW Sampling Procedure

If participant declined, they were replaced by another randomly selected participant from the same ward to maintain the calculated sample distribution and mitigate non-response bias. Detailed sampling techniques are illustrated in Figure 1.

### Data Collection Procedure

Data collection was meticulously carried out using a structured, interviewer-administered questionnaire, which was initially developed in English and rigorously translated into Swahili with three rounds of linguistic checks to ensure accuracy and clarity for participants. This questionnaire was structured using questions from existing literature,^12,15,23^ and validated questions on contraceptive use from the Tanzania Demographic and Health Survey (TDHS).^24^ Trained research assistants were responsible for administering the questionnaire and completing survey forms for each FCSW, with each participant receiving 5,000 Tanzanian shillings (approximately 2 USD) as an incentive. The questionnaire's validity was established through several psychometric steps, including face validity where experts and psychometricians reviewed questions for clarity and common errors and content validity, where a panel of experts assessed the representativeness of items to the theoretical construct. A pilot test on 16 respondents identified areas for improvement and provided initial response distribution, followed by data cleaning, reverse-coding of negative questions. Data from pilot study were not involved in final analysis.

Ensuring high data quality was paramount, with measures implemented throughout the collection process including automated and high-frequency checks like range, logic, and consistency checks to identify and report issues. Duplicate responses and conflicting answers were checked, and survey completion times were monitored to assess engagement. Comprehensive research assistant training, vital for minimizing interviewer effects and reducing non-sampling errors, involved sessions on study background, respondent selection, effective interviewing, questionnaire completion, and techniques like active listening. Regular quality reviews of completed questionnaires and weekly feedback ensured consistent application of protocols and real-time correction of data quality issues.

### Data Processing and Analysis

Data were collected using paper-based structured questionnaires, which were then manually entered into Statistical Package for Social Sciences (SPSS) version 29 for processing and analysis. Before data analysis commenced, a comprehensive data cleaning process was undertaken to identify and rectify any duplicate, erroneous, or missing entries. This rigorous quality check ensured the integrity and validity of the dataset. Descriptive statistics were subsequently generated to summarize the broad socioeconomic and demographic characteristics of the study participants, presented as frequencies and percentages. Participant age was categorized into three distinct groupings to facilitate further analysis. Logistic regression analysis was employed to investigate the relationship between emergency contraception use (the primary outcome variable) and various explanatory variables. Both univariate and multivariate logistic regression models were utilized to assess these associations, providing a comprehensive understanding of the factors influencing emergency contraception use.

### Ethical Consideration

The ethical conduct of this study was paramount, especially given the vulnerable nature of our participant population. We obtained full ethical clearance from The University of Dodoma's Institutional Review Board, ensuring adherence to national and international guidelines for human subject's research. Furthermore, approval was secured from the Regional Administrative Secretary and local authorities, acknowledging the community context of our data collection. All research assistants underwent rigorous, in-depth training on protecting human subjects, emphasizing the critical importance of maintaining participant confidentiality and privacy. Each participant provided informed consent using institutionally approved forms, clearly understanding their right to voluntarily participate or withdraw without penalty, thereby fully safeguarding their autonomy. The unique ethical complexities of researching female commercial sex workers, including potential risks of stigma or discrimination, were proactively addressed by implementing stringent data anonymization protocols, such as using anonymous survey numbers, and by creating a safe, non-judgmental environment for data collection.

## RESULTS

### Sociodemographic Characteristics

Three hundred twenty six commercial sex workers were included in this study, with a response rate of 85%. The majority of study respondents, 58%, were aged between 21 to 31 years, 85.2% were not living with a male partner, 62.3% began sexual intercourse between 15 years to 20 years, 88.1% had post-primary education, and 52.8% had family. Among all participants, 51.8% experienced sexual assault by being forced to perform sexual intercourse (Table 1).

**TABLE 1:** Sociodemographic Characteristics of Study Respondents

Variable	Frequency	Percentage
Age group		
21−30	189	58
31 and above	64	19.6
Marital Status		
Married	48	14.7
Single	149	45.7
Divorced	109	33.4
Widow	20	6.1
Religion of participants		
Christian	176	54
Muslims	131	40.2
No religion	14	4.3
Other religions	5	1.5
Age to begin the sex		
Below 14	3	0.9
15−20	203	62.3
21−30	111	34
Above 30	9	2.8
Use of drug abuse		
Yes	174	53.4
No	152	46.6
Level of education		
Standard seven	39	12
Form four lever	145	44.5
Form six lever	104	31.9
University level	38	11.7
Having family		
Yes	172	52.8
No	154	47.2
Force to sex		
Yes	169	51.8
No	157	48.2

### Factor Associated With Emergency Contraceptive Use Among Commercial Sex Workers

The study found that a significant majority of the respondents (64.4%) had never used EC. Multivariate logistic regression analysis identified several key factors independently associated with EC use. Education level showed an inverse relationship, with those having ordinary secondary education (AOR=0.37, 95% CI=0.16–0.88, *P*=.024) and college-level education (AOR=0.13, 95% CI=0.04–0.47, *P*=.002) being significantly less likely to use EC compared to those with no formal education. Furthermore, a history of drug use was strongly associated with a reduced likelihood of EC use (AOR=0.35, 95% CI=0.19–0.61, *P*<.001). Conversely, prior awareness of emergency contraceptives emerged as a powerful predictor of use, with those who had ever heard about EC being substantially more likely to use them (AOR=6.20, 95% CI=3.44–11.17, *P*<.001) Table 2.

**TABLE 2:** Factor Associated with Emergency Contraceptive Use Among Commercial Sex Workers

Variable	OR (95% CI)	*P value*	AOR (95% CI)	*P value*
Level of Education				
Primary and below	1			
O'level secondary	0.63 (0.31−1.27)	.195	0.37 (0.16−0.88)	.024
A'level secondary	0.61 (0.29−1.28)	.187	0.44 (0.18−1.05)	.065
College and above	0.16 (0.05−0.49)	.001	0.13 (0.04−0.47)	.002
Ever Abuse Drugs?				
No	1			
Yes	3.73 (2.28−6.11)	<.001	0.35 (0.19−0.61)	<.001
Ever Being Pregnant?				
No	1			
Yes	2.11 (1.30−3.42)	<.001	1.43 (0.81−2.50)	.218
Reproductive education about EC				
No	1			
Yes	5.25 (3.15−8.76)	<.001	6.20 (3.44−11.17)	<.001

OR=Odds Ratio, AOR=Adjusted Odd Ratio, CI=Confidence Interval, EC=Emergency contraceptive

## DISCUSSION

This study aimed to assess the FCSW's utilization of emerging contraceptives and its contributing factors. According to the results of our survey, the majority of FCSW did not use emergency contraception. Low EC consumption has been explained by various factors, such as social and religious restrictions, ignorance, and some people being less cautious.^25^ The use of emergency contraceptives was minimal, as reported in several studies that share the same conclusions as this study's findings.^26^ Contrary to our findings, however, several studies have demonstrated that FCSWs are aware of emergency contraceptives and have been using them.^27^ Therefore, notwithstanding the difficulties in performing commercial sex, our findings represent a difficulty in imparting EC knowledge to workers in low resource contexts.

The multivariate logistic regression analysis revealed several significant factors associated with EC use among FCSW. These findings present both alignments and divergences with previous research, highlighting areas for targeted interventions.

The study found that FCSW with higher educational attainment, specifically those with ordinary secondary education and college level education, were less likely to use emergency contraceptives. This finding contrasts with other studies that generally show increased EC use with higher education levels among women who have ever had sex.^28^ Similarly, a study in Brazil reported that having a high level of education was positively associated with EC use.^29^ This discrepancy suggests that while higher education may increase general knowledge, it might not translate into increased EC utilization within the specific context of female commercial sex workers in this study, possibly due to other confounding factors or misconceptions. Health policymakers and educators should prioritize the integration of comprehensive and accurate EC education into both formal and informal educational curricula, particularly in rural and underserved areas. These educational programs should clarify common misconceptions about EC, emphasize appropriate timing and effectiveness, and address specific concerns that might deter highly educated individuals from using EC effectively.^30^

The study indicated that ever having used drugs was negatively associated with EC utilization among FCSW. This suggests that drug use may be a barrier to consistent EC use. This finding aligns with research indicating that drug use can be a significant risk factor for various sexual and reproductive health issues, often leading to inconsistent contraceptive practices.^31^ Another studies on female sex workers in Vietnam and on the US-Mexico border highlight a high prevalence of drug use and its association with increased risk behaviours, which could indirectly hinder effective contraceptive use.^32^ Integrated health services are crucial, combining reproductive health counselling with substance abuse support programs. These interventions should aim to reduce drug use and simultaneously improve access to and education about EC for female sex workers who use drugs, recognizing that this population may have unique challenges in accessing and utilizing healthcare services. Training healthcare providers to offer non-judgmental counselling is also vital.^30^

A strong positive association was found between having ever heard about emergency contraceptives and their utilization. This reinforces the critical role of awareness in promoting EC use, a finding consistently supported by numerous studies in a global scoping review.^7^ However, awareness does not always translate to correct knowledge or utilization, as demonstrated by findings where many who had heard of EC still lacked understanding of its correct timing or effectiveness.^23^ Health promotion campaigns should continue to increase awareness of EC, utilizing diverse channels such as health workers, social media, and community outreach programs. The campaigns should not only focus on general awareness but also on providing detailed, accurate information regarding the correct timing, methods, and benefits of EC to bridge the gap between awareness and appropriate utilization among FCSW.

### Strengths and Limitations

This study offers valuable insights into the factors influencing emergency contraceptive use among FCSW, a population often underserved and under-researched. A notable strength is the use of multivariate logistic regression, which allowed for the identification of independent predictors of EC use while controlling for confounders. The study's focus on a specific vulnerable group also fills a critical gap in the literature. However, this study also has several limitations. The cross-sectional design prevents the establishment of causal relationships. Additionally, the reliance on self-reported data may be subject to recall bias or social desirability bias, potentially affecting the accuracy of reported drug use and contraceptive practices. The limited comparison with similar studies from the immediate region and the lack of in-depth discussion on cultural and contextual factors may also restrict the generalizability of these findings to other settings.

### Recommendations for Tanzania Health Policy and Further Research

Based on these findings, this study recommends several actions for Tanzania's health policy and future research. For health policy, it is imperative to develop and implement targeted, culturally sensitive educational programs for female commercial sex workers that address misconceptions about EC and its proper use, particularly among those with higher education. These programs should be integrated into broader sexual and reproductive health services and, crucially, combined with harm reduction strategies and substance abuse support services to address the negative association between drug use and EC uptake. For further research, we recommend conducting longitudinal studies to explore the causal pathways between educational attainment, drug use, and EC utilization. Future studies should also employ qualitative methods to delve deeper into the cultural and structural barriers to EC access and use among this population, especially in diverse regional contexts within Tanzania. It is also important to investigate the specific reasons why higher education does not correlate with increased EC use in this specific group.

## CONCLUSION

In contrast to the general population, the use of emergency contraceptives among FCSW is still low for various reasons. The findings underscore the complex interplay of educational attainment, drug use, and awareness in shaping emergency contraceptive utilization among female commercial sex workers. While awareness is a strong facilitator of EC use, higher education levels surprisingly showed an inverse relationship with EC use in this specific population, contrasting with general population trends. This suggests that targeted interventions are needed to address the unique barriers faced by this vulnerable group, including misconceptions among the educated and the compounding challenges posed by drug use. Therefore, a multi-faceted approach combining comprehensive education, integrated health services, and sustained awareness campaigns is essential to improve EC access and utilization for female commercial sex workers.
